# Coordination of Chloroplast Activity with Plant Growth: Clues Point to TOR

**DOI:** 10.3390/plants11060803

**Published:** 2022-03-17

**Authors:** Stefano D’Alessandro

**Affiliations:** 1 Dipartimento Scienze della Vita e Biologia dei Sistemi, Università degli Studi di Torino, 10135 Torino, Italy; stefano.dalessandro@unito.it; 2 CNRS, CEA, BIAM UMR7265, Aix Marseille University, F-13009 Marseille, France

**Keywords:** chloroplast, stress response, TOR, photooxidation, plant growth

## Abstract

Photosynthesis is the defining function of most autotrophic organisms. In the plantae kingdom, chloroplasts host this function and ensure growth. However, these organelles are very sensitive to stressful conditions and the photosynthetic process can cause photooxidative damage if not perfectly regulated. In addition, their function is energivorous in terms of both chemical energy and nutrients. To coordinate chloroplast activity with the cell’s need, continuous signaling is required: from chloroplasts to cytoplasm and from nucleus to chloroplasts. In this opinion article, several mechanisms that ensure this communication are reported and the many clues that point to an important role of the Target of Rapamycin (TOR) kinase in the coordination between the eukaryotic and prokaryotic sides of plants are highlighted.

## 1. Why Plants Need to Regulate Chloroplasts Activity

Green is the color that everybody associates with the plant kingdom but, only recently, Arp et al., explained the dominance of this color in photosynthetic organisms [1]. According to their model, the best wavelengths to absorb are in the red and blue portion of the spectrum, reflecting part of the more intense green, as chlorophylls do. It appears that the photosynthetic machinery evolved not for maximum light harvesting but rather for maximum efficiency, avoiding photooxidation [1].

In the plantae kingdom, chlorophylls are found in the antennae complexes in the thylakoid membranes in chloroplasts. In antennae, light harvesting complexes (LHCI and LHCII) concentrate light energy on the photosystems (PSI and PSII). In addition, plastoquinone and the cytochrome b6f participate in the photosynthetic electron transport chain (PETC). All of these elements ensure electron flow between the photosystems that are physically separated, PSII complex being mostly in the stacked grana domains of the thylakoid and PSI in the unstacked lamellar regions [2]. It is critical to energetically couple the two photosystems to achieve the reduction of nicotinamide adenine dinucleotide phosphate (NADP+) to NADPH and subsequent carbon dioxide (CO_2_) fixation (carbon assimilation) [3].

In addition, the light source is not constant and varies greatly, from 0 photon flux density (PFD) up to 2000 PFD, with a diurnal rhythm and seasonal variation. It may also be very fast due to shading [4]. Several processes enable the management of electron fluxes in the PETC to keep the photosystems coupled, such as cyclic electron flow and non-photochemical quenching (NPQ). Under the term of NPQ, several processes have been grouped and divided between processes that do not lead to thermal energy dissipation, such as chloroplast movement (qM) and state transition (qT), and processes that lead to thermal-energy dissipation, such as PsbS-dependent quenching (qE), photo-inhibitory quenching (qI), Zeaxanthin-dependent quenching (qZ), and sustained quenching (qH) [5]. 

Although light capture is finely tuned, light can be absorbed in excess to its use in photosynthesis [6,7]. This excess light condition does not require high light intensities, being dependent on the availability of energy sinks [4,8]. Indeed, NPQ is already active at low PFDs and a leaf energy balance model calculated that only the minority of absorbed light is used for CO_2_ fixation and sugar production [7]. Altered electron flow between photosystems, e.g., related to a lack of water, the ubiquitous electron donor, or slowed metabolism causing a lack of energy sinks, such as CO_2_ fixation, can put the plant in a state of excessive light, even at low PFD [9]. This is the case with stressful conditions, such as drought and cold, which lead to the excessive energy pressure on photosystems and subsequent generation of reactive oxygen species (ROS) [10,11]. PSII is very sensitive to ROS, especially singlet oxygen, which is generated very early under stress conditions, and photodamage and repair of D1 constitute a photoprotective mechanism in itself to protect PSI [12,13]. 

Sometimes, carbon assimilation may be necessary even if the conditions make photosynthesis dangerous, and plants, unable to escape the sustained ROS production, may suffer photooxidative stress and cell death [8,14]. In particular, when ROS accumulate, they can react with and damage many biomolecules (carotenoids, nucleic acids, amino acids, lipids, and possibly sucrose) [13,15]. In addition to direct toxicity, some oxidative by-products of lipids, such as reactive carbonyl species (RCS), are toxic to the cell and can induce plant cell death [16,17]. 

At the same time, ROS also play a signaling role and communicate the photosynthetic state to the plant cell by taking part in the operational retrograde signaling [18,19]. Hydrogen peroxide (H_2_O_2_) has been reported to move directly from the chloroplasts to the nucleus and chloroplasts are often associated with the nucleus under stress conditions [18,20]. Singlet Oxygen can directly oxidize the proteins Executer 1 (EX1) and Executer 2 (EX2) and the equilibrium between the two oxidation modulates retrograde signaling [21,22]. 

In addition, oxidative by-products of biomolecules also elicit nuclear responses. Among these, β-cyclocitral is a very early indicator of PSII damage and, pretreatment with this volatile molecule induces a photoprotective state in plants [23,24]. β-cyclocitral, RCS, and molecules such as 3′-phosphoadenosine-5′-phosphate (PAP) and methylerythritol cyclodiphosphate (MeCPP) trigger a nuclear response to reduce photooxidation [25,26,27]. In particular, the link between ROS production and apocarotenoid generation at PSII and RCS is so close that these molecules share the same catabolic/detoxifying enzymes [19,28]. 

Even when environmental conditions are optimal, due to the much higher concentration of oxygen (O_2_, 21%) than CO_2_ (0.4%) in the atmosphere, the Ribulose-1,5-bisphosphate carboxylase/oxygenase (Rubisco) catalyzes one O_2_ every three CO_2_ molecules in the photorespiration process [29]. The recycling of the photorespiration product 2-phosphoglycerate (2-PG) back to 3-PG is a wasteful process due to the consumption of stromal ATP and the generation of H_2_O_2_ [30,31].

In addition to the dangerous production of ROS, chloroplast function is energivorous in terms of both chemical energy, consuming most of the ATP and nutrients it produces during photosynthesis [7,32]. Indeed, Rubisco is by far the most abundant protein on Earth [33]. Thus, modulating chloroplast activity is not only necessary to avoid photooxidation but also essential to save and remobilize important nutrients. 

## 2. Mechanisms Regulating Chloroplasts Activity

Although millions of years have passed since the inclusion of chloroplasts in the eukaryotic cell, they remain rather independent organelles that require continuous signaling to be coordinated with the needs of the organism. In addition to the examples of retrograde signaling mentioned above, biogenetic retrograde signaling based on the tetrapyrrole/genomes uncoupled (GUN) mechanism has been recently reviewed [34].

At the same time, a very strong transfer of genes from the organelle to the nucleus, leaving about 5% of the original genome, has allowed an improvement in energy efficiency at the price of a strong dependence on the nuclear genome [35]. More than 90% of the proteins in the chloroplast are encoded in the nucleus, and the most characteristic chloroplast functions, such as photosynthesis and carbon fixation, require close coordination between the chloroplasts and nucleus [36]. Clear examples are antenna complexes, in which cores encoded in the chloroplast are surrounded by LHCs encoded in the nucleus, and the large Rubisco subunit (LSU) encoded in the chloroplast, which is a holoenzyme (8 LSU and 8 SSU) with the small Rubisco subunits encoded in the nucleus (SSU) [37]. 

Nuclear-encoded chloroplast genes (NECGs) are transcribed in the nucleus by the canonical RNA polymerase II, then the mRNA is transported from the nucleus to the cytosol and translated by the ribosomes [38]. 

This mechanism allows a first level of control of chloroplast activity by the plant cell (the so called “anterograde signaling”) based on the regulation of transcription and translation of NECGs. This level of control can be achieved through cis-acting enhancer elements associated with photosynthesis, such as the Light Response Elements (LRE), including GT elements, G-Box elements, I-Box elements, Gap-Box elements, AT-rich elements, GC-rich elements, and L-Box elements [39]. However, although several LREs and their binding proteins have been identified, no single element has been shown to confer light reactivity, suggesting that a complex combination of cis-acting sequences is required to confer the correct photo-reactivity to promoters [40].

An enormous role in the control of nuclear transcription by light is played by phytochromes, cryptochromes, and phytochrome interacting factors (PIF) [41,42,43,44]. Photoreceptors are involved in all major functions of plant biology and, while they were initially described as chloroplast biogenic factors, several works are now demonstrating their involvement in the response to stress conditions [45,46,47]. In addition, photoreceptors can also alter promoter selection by RNA polymerase in the nucleus to modify the N-terminus of proteins and their subcellular localization [48]. 

Pre-proteins synthetized in the cytosol can be finally imported into the chloroplast through recognition of an N’-terminal Chloroplast Targeting Peptide (CTP) via the Tic and Toc translocon complexes, discussed below [49,50]. 

Among these proteins are the regulators of organelle gene expression (ROGEs), which can directly alter transcription in the chloroplast. The first is the nuclear-encoded plastidial RNA-polymerase (NEP), which ensures plastidial transcription together with the plastidial-encoded polymerase (PEP) [51]. However, most plastidial transcription units are preceded by multiple promoters, allowing transcription by PEP as well as NEP [52]. Six sigma transcription factors are present in *Arabidopsis thaliana*, all encoded in the nuclear genome and can confer promoter selectivity, thus altering plastidial transcription [53,54].

The signaling of the unusual nucleotide guanosine-3,5-(bis)diphosphate (ppGpp) is also of interest, which may act as a proliferation brake in prokaryotes and whose biosynthetic pathway, mediated by RelA-SpoT homologue (RSH) proteins, has been remobilized to the nucleus [55,56]. Thus, although ppGpp is only metabolized in the chloroplast, both the synthetases and hydrolases that allow fine control of ppGpp homeostasis must be imported [57,58]. This is particularly important under stress conditions, such as nitrogen deprivation or virus infection, where ppGpp accumulation plays an important role [59,60,61].

In addition, several nuclear-encoded proteins are involved in the chloroplastic RNA processing. Examples are pentatricopeptide (PPR) RNA-binding proteins, which, contributes to the stability and editing of specific RNAs in the chloroplast, CRS1–YhbY (CRM) domain, and plant organelle RNA recognition (PORR) domain proteins [62,63,64].

## 3. Chloroplastic Import: A Dynamic Gatekeeper of Coordination?

Plants must achieve flawless coordination between chloroplast and nuclear functions to avoid photooxidation and optimize nutrient economy. A key step in this process is the regulation of chloroplast import, especially under stress conditions, which induces extensive changes in the plastidial proteome [50]. Most of the chloroplast proteins are synthesized in the cytosol as pre-proteins, still possessing the CTP, and must pass through the chloroplast double membrane to reach their functional destination. 

Cytosolic chaperones bind to the pre-proteins, facilitate their navigation to the organelle, and maintain an unfolded conformation suitable for import [65]. Hsp90 together with Hsp70-Hsp90-organizing protein (Hop) and the immunophilin FK506-binding protein 73 (FKBP73) has been proposed to transport pre-proteins to the outer envelope membrane (OEM) [66]. Alternatively, HSP70 with chaperones 14-3-3 has also been implicated in the delivery of phosphorylated pre-proteins to the translocation complexes, where they are dephosphorylated prior to the import [67,68]. 

The import of pre-proteins into the chloroplast is mainly controlled by two multi-protein complexes, the translocon at the outer chloroplast membrane (TOC) and the translocon at the inner chloroplast membrane (TIC) [69]. In particular, at the level of the TOC complex, Toc33 and Toc159, on the cytosolic side, are involved in the substrate recognition while Toc75 constitutes the pre-proteins entry channel. 

Importantly, chloroplast import responds to developmental cues and stress conditions, and its own components may be targeted for degradation by the proteasome or oxidated by ROS [70,71,72]. In response to developmental or environmental cues, the suppressor of ppi1 locus 1 (SP1) promotes the degradation of TOC complexes, thereby suppressing the import of plastidial pre-protein. The sensitivity (and increased H_2_O_2_ accumulation) of sp1 mutant lines to stress conditions and the resistance of the SP1 overexpressors suggest that chloroplast gate closure, through TOC complex degradation, is a key mechanism to reduce energy pressure on the photosynthetic chain and to cope with stressful environmental conditions [73]. 

A direct consequence of deregulation of chloroplast import is an increase in the presence of pre-proteins in the cytosol. To avoid overcrowding, chloroplast-targeted pre-proteins can be marked for ubiquitin–proteasome (UPS)-mediated degradation, which is another process in the cytosolic control of chloroplast function [74]. This process involves the HSP70 isoform Hsc70-4, which interacts with the Targeting Peptide of pre-proteins, recruiting them to the C-terminus of Hsc70-interacting protein (CHIP) E3 ligase for ubiquitination and degradation by the 26S Proteasome [75]. In addition, the ubiquitin proteasome system has also been implicated in the regulation of Golden2-like 1 transcription factor (GLK1), which promotes chloroplast activity and biogenesis and is degraded by the proteasome in response to chloroplast stress, probably through GUN1 retrograde signaling [76].

Finally, when a stress stimulus arrives, there is only a short window of time to modify and import nuclear-encoded proteins into the chloroplast before the gate closes. Protein phosphorylation is a very fast reaction occurring on a time scale of seconds to minutes, which has been observed in the regulation of TOC import capacity and of SSU import into the chloroplast [77,78,79,80,81]. Indeed, Serine/threonine/tyrosine (STY) protein kinases have been proposed to phosphorylate the SSU CTP to regulate the import of the related pre-protein [67]. Interestingly, the amino acid isoleucine can bind to the ACT domain of STY kinases and module their activity, establishing a link between nutrient availability and SSU import into the chloroplast [79]. In addition, purple acid phosphatase 2 (PAP2) activity has been shown to be required for chloroplast import [78,79,81]. 

## 4. Nutrients/Metabolites Exchange as Signaling

Although highly dependent on nuclear regulation, chloroplasts are highly compartmentalized organelles in the plant cell. Not only are they separated from the cytosol by a double membrane system (outer and inner membrane), but they also have an internal membrane system, the thylakoids, which allows photosynthetic reactions. 

Like proteins that require an import system to enter the chloroplast, nutrients, solutes, and metabolites move between the cytosol and the organelles via a very rich set of channels and transporters [82]. 

Maintaining optimal ion concentrations within the chloroplast is critical for pH regulation, chloroplast volume, thylakoid staking, and proper photosynthetic reactions [83]. Therefore, sodium, potassium, chloride, calcium, and magnesium as well as iron, manganese, and copper must be imported into the chloroplast [83,84]. 

At the same time, the most intuitive metabolite flux from the chloroplast is the efflux of phosphorylated carbohydrates and reducing equivalents (dihydroxyacetone phosphate and malate), but chloroplasts are also unique sites for the biosynthesis of fatty acids and nine amino acids [7,31,85,86]. In addition to the many substrate-specific transporters of the inner envelope, the outer envelope is enriched in five proteins with transport functions: OE Porins (OEP 16, 21, 24, and 37) and an ATP-binding cassette (ABC) transporter, which show different degrees of specificity towards substrates [82,87,88]. Thus, metabolite flux appears to be tightly controlled and may consequently constitute signaling. Indeed, triose phosphate efflux has been implicated in the very fast signaling of excessive light (less than 1 min), the phosphate/trioso phosphate translocator (*tpt*) mutant being fully deregulated in the response of four *Apetala2/Ethylene responsive factor* (*AP2/ERF*) marker genes [85]. More importantly, glucose and glutamine can be exported from the chloroplast to the cytosol. 

Sugar and nitrate sensing is primitive and involves proteins that have often been conserved during evolution [86]. Indeed, many players are involved in these primordial pathways, such as the Nitrate transporter 1 (NRT1) transporter for nitrate and the sucrose efflux transporter (SWEET) proteins and the Hexokinase (HXK1) for sugars. Their intricate pathways have recently been reviewed [86]. At the same time, the TOR kinase and sucrose non-fermenting 1 (SNF1)-related kinase 1 (SnRK1) are recognized as key regulators of eukaryotic nutrient sensing [86]. 

## 5. Mutual Regulation of TOR and Chloroplast Activity 

In eukaryotes, two kinase complexes have been shown to play a fundamental and conserved role in nutrient signaling: SnRK1 and TOR. 

TOR associates with other proteins to form the TORC1 complex in plants. The main components are the TOR kinase, regulatory-associated protein of mammalian TOR (Raptor), and lethal with SEC13 protein 8 (LST8). TORC1 promotes cell growth in response to nutrient availability and integrates nitrogen and carbon signals. 

Nitrates (NO^3−^), ammonium (NH^4+^), and Glutamine all induce TOR activity although amino acids generated by plant-specific pathways (Glutamine, Cysteine, and Glycine) have the greatest activation potency [87,88]. In particular, these nitrogen sources activate the small GTPase Rho-related proteins (ROP2) that bind and activate TOR [87,89]. 

SnRK1 is a multi-protein complex that includes a kinase α subunit and two regulatory β and βγ subunits [90]. Its activity is induced by energy deprivation and repressed by sugars, including glucose 6-phosphate and trehalose 6-phosphate in plants. Among other activities, SnRK1 can interact with and phosphorylate RAPTOR1B in vivo and in vitro, inhibiting TOR activity [91,92,93].

In addition, stress and ABA activate SnRK2 and enhance the activity of SnRK1, which phosphorylates RAPTOR and inhibits TOR activity [94,95]. ABA is a phytohormone common to several stress responses, which is also rapidly accumulated under excessive light, due to the induction of the 9-cis epoxycarotenoid dioxygenase (NCED), the first dedicated step in the chloroplastic biosynthesis of ABA [96,97]. 

Finally, chloroplasts have a very strong control over plant growth through TOR regulation, especially in light of the recent demonstration that photosynthetic carbon assimilation has a direct impact on TOR activity in *Chlamydomonas reinhardtii* [98]. 

On the other hand, TOR can also influence chloroplast activity. It has been known for several years that suppression of TOR activity in Arabidopsis reduces greening and expansion of cotyledon, photosynthesis, chlorophyll biosynthesis, light reactions, and CO_2_ fixation [99]. A decrease in electron transport rate and chlorophyll concentration, an increase in NPQ, and alterations in antennae distribution between photosystems were also observed in *C. reinhardtii* after TOR inhibition [100]. 

How can TOR influence the amount of chlorophyll and regulate chlorophyll fluorescence and NPQ? One possibility is through chlorophagy. TOR is a well-known repressor of autophagy in plants [93,101,102]. Indeed, the autophagy related proteins ATG101, ATG1a, ATG1b, ATG1c, and ATG13 have been found in the interactome of TORC1 in Arabidopsis, and ATG1 and ATG13 have been proposed as direct phospho-target of TOR [103]. Chlorophagy and Rubisco containing bodies, two chloroplast recycling pathways, are ATG-dependent processes. However, so far there is no evidence that TOR directly affects these pathways [104,105,106]. 

Alternatively, TOR could regulate the transcription and the translation of important nuclear-encoded elements in the chlorophyll biosynthetic pathway and for PETC. Indeed, repression of photosynthesis-associated genes, involved in chlorophyll biosynthesis, light reactions, and CO_2_ fixation was observed in experiments that inhibited TOR activity for 24 h [99,107]. Is there a role for TOR in GLK regulation and retrograde signaling?

In an integrated transcriptomic and proteomic approach, it was observed that TOR inhibition by Torin2 represses the translation of several (20 to 30%) chloroplastic mRNAs, in less than 2 h, with a corresponding decrease in chlorophyll levels [108]. 

At the same time, a short inactivation of TOR (2 h of Torin2) does not affect the expression of photosynthesis-associated nuclear genes, in contrast to prolonged inhibition of TOR activity, which strongly represses the expression of photosynthesis-associated nuclear genes (PhANGs) [99,108]. Interestingly, the authors suggest that TOR inactivation first represses translation in the chloroplast and that this secondarily leads to repression of PhANG expression via retrograde signaling [108,109]. In line with this hypothesis, GLK1 expression was slightly repressed in their analyses, suggesting that repression of PhANGs would follow.

Finally, TOR has also been implicated in the regulation of translation of nuclear-encoded mRNA in response to light required for cotyledon opening, through phosphorylation of ribosome protein 6 (RPS6), in a pathway dependent on phytochrome, constitutive photomorphogenesis 1 (COP1), and auxin [110]. 

## 6. Conclusions and Open Questions

**Figure 1:**
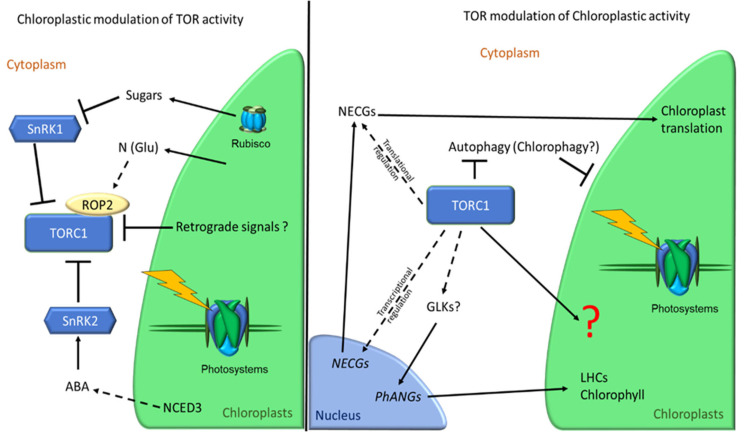
Reciprocal regulation between TOR and chloroplast activity. Chloroplast activity influences the activity of the TOR complex (TORC1) through the production of sugars (glucose and trehalose 6-phosphate), through the release of amino acids in the remobilization of carbon and nitrogen (glutamine) and through the biosynthesis of phytohormones (ABA). On the contrary, TORC1 activity influences different aspects of chloroplast physiology (translation, photosynthetic efficiency, chlorophyll concentration) by still unknown mechanisms, which might involve the transcriptional and translation control of NECGs (including PhANGs) and the regulation by phosphorylation of ATG1 and ATG13, involved in autophagy and probably in chlorophagy.

Coordination between the chloroplast and the nucleus must be impeccable to avoid photooxidation, and several checkpoint mechanisms are present in plants. Continuous retrograde signaling communicates photosynthetic status to the cell and elicits a measured response from the nucleus. The products of chloroplast activity may be central to the coordination of chloroplast function and plant growth converging on the TOR kinase. Yet the mechanism(s) by which TOR controls chloroplast activity, as well as the influence of retrograde signals on TOR itself, remain major unanswered scientific questions. A schematic summary of the reciprocal regulation between TOR and chloroplast activity can be found in Figure 1.

## Data Availability

Not applicable.
